# Complete Genome Sequence of Early Passaged Human Herpesvirus 6A (GS Strain) Isolated from North America

**DOI:** 10.1128/genomeA.00012-13

**Published:** 2013-06-13

**Authors:** Annie Gravel, Dharam Ablashi, Louis Flamand

**Affiliations:** Division of Infectious Diseases and Immunity, CHU de Quebec Research Center, Quebec, Canadaa; HHV-6 Foundation, Santa Barbara, California, USAb; Department of Microbiology, Infectious Disease and Immunology, Faculty of Medicine, Université Laval, Quebec, Canadac

## Abstract

The genome sequence of the original human herpesvirus 6A (HHV-6A) (GS isolate) has never been determined. DNA from a very low passage (<5 times) HHV-6A GS stock was isolated and sequenced, making it the first HHV-6A of U.S. origin to be fully sequenced. Such a sequence will be helpful for comparative analysis with other HHV-6A strains, including the U1102 strain isolated from Uganda.

## GENOME ANNOUNCEMENT

The human betaherpesvirus subfamily is composed of 4 viral species: cytomegalovirus and the herpesvirus (HHV)-6A, HHV-6B, and HHV-7. The original HHV-6 isolate GS belongs to HHV-6A and was first isolated in the United States in 1986 from subjects with lymphoproliferative disorders and AIDS patients (1). Subsequently, a second HHV-6A isolate from Uganda, U1102, was reported (2). Complete genome sequencing of U1102 was reported in 1995 (3). For reasons that are not clear, the entire sequence of HHV-6A GS was never determined. Considering that strains GS and U1102 are from very different geographical regions and that a complete genome sequence from a North American isolate is lacking, it became important to obtain the entire genomic sequence of the original GS isolate, considering that HHV-6A is the type species of the genus.

HHV-6A GS has been and continues to be extensively studied by researchers. Over the last 26 years, HHV-6A GS has been passaged countless number of times in tissue culture (either in activated primary T cells, HSB-2, or SupT1 cell lines). Knowing that extended tissue culture passages might alter the genetic content of the viruses, it was important to have access to a low-passaged HHV-6A GS isolate. We were fortunate to retrieve an aliquot of HHV-6 GS that was passaged only 4 times in cord blood mononuclear cells. From this stock of virus, we isolated the linear viral DNA. The viral DNA was sequenced using an Illumina genome analyzer (Illumina, Inc.). Gap filling was carried out by using standard PCR and subsequent Sanger sequencing. The whole-genome sequence was analyzed using Newbler assembler 2.3 (Roche). Open reading frames (ORFs) were predicted by GenMark.hmm (4), and genes were annotated by comparison with HHV-6A U1102 (GenBank accession no. NC_001664) and homology searches of GenBank (5).

The HHV-6A GS genome is composed of 156,885 nucleotides and 88 putative genes, of which 12 contain multiple exons. In comparison, the HHV-6A GS genome is 2,437 nucleotides shorter than that of HHV-6A U1102. Overall nucleotide identities with HHV-6A U1102 and HHV-6B Z29 are 98% and 89%, respectively. In comparison with HHV-6A U1102 and HHV-6B Z29, the most dramatic variations observed consist of a reduction in the number of repeats within the R2 and R3 internal repeated regions.

### Nucleotide sequence accession number.

The human herpesvirus 6A (GS isolate) nucleotide sequence has been deposited in GenBank under sequence accession no. KC465951.
